# Daily inhalation of hydrogen gas has a blood pressure-lowering effect in a rat model of hypertension

**DOI:** 10.1038/s41598-020-77349-8

**Published:** 2020-11-26

**Authors:** Kazuhisa Sugai, Tomoyoshi Tamura, Motoaki Sano, Shizuka Uemura, Masahiko Fujisawa, Yoshinori Katsumata, Jin Endo, Joe Yoshizawa, Koichiro Homma, Masaru Suzuki, Eiji Kobayashi, Junichi Sasaki, Yoji Hakamata

**Affiliations:** 1 Department of Basic Science, Nippon Veterinary and Life Science University, School of Veterinary Nursing and Technology, Tokyo, 180-8602 Japan; 2 Department of Emergency and Critical Care Medicine, Keio University School of Medicine, Tokyo, 160-8582 Japan; 3 The Center for Molecular Hydrogen Medicine, Keio University, Tokyo, 108-8345 Japan; 4 Department of Cardiology, Keio University School of Medicine, Tokyo, 160-8582 Japan; 5 Department of Emergency Medicine, Tokyo Dental College Ichikawa General Hospital, Chiba, 160-8582 Japan; 6 Department of Organ Fabrication, Keio University School of Medicine, Tokyo, 160-8582 Japan

**Keywords:** Hypertension, Chronic kidney disease

## Abstract

A recent clinical study demonstrated that haemodialysis with a dialysate containing hydrogen (H_2_) improves blood pressure control in end-stage kidney disease. Herein, we examined whether H_2_ has a salutary effect on hypertension in animal models. We subjected 5/6 nephrectomised rats to inhalation of either H_2_ (1.3% H_2_ + 21% O_2_ + 77.7% N_2_) or control (21% O_2_ + 79% N_2_) gas mixture for 1 h per day. H_2_ significantly suppressed increases in blood pressure after 5/6 nephrectomy. The anti-hypertensive effect of H_2_ was also confirmed in rats in a stable hypertensive state 3 weeks after nephrectomy. To examine the detailed effects of H_2_ on hypertension, we used an implanted telemetry system to continuously monitor blood pressure. H_2_ exerted an anti-hypertensive effect not only during daytime rest, but also during night-time activities. Spectral analysis of blood pressure variability revealed that H_2_ improved autonomic imbalance, namely by suppressing the overly active sympathetic nervous system and augmenting parasympathetic nervous system activity; these effects co-occurred with the blood pressure-lowering effect. In conclusion, 1-h daily exposure to H_2_ exerts an anti-hypertensive effect in an animal model of hypertension.

## Introduction

Molecular hydrogen (H_2_) is a versatile gas with antioxidant and anti-inflammatory properties and no apparent side effects^1^. We previously demonstrated in animal models that H_2_ inhalation is a promising therapeutic option for ischaemia–reperfusion injury in emergency and critical care settings such as acute myocardial infarction, cardiac arrest, and haemorrhagic shock^2–5^. We also conducted a proof-of-concept clinical study to validate the efficacy and safety of H_2_ gas inhalation therapy for patients with acute myocardial infarction and post–cardiac arrest syndrome^6,7^. A multicentre, double-blind, randomised controlled trial is currently underway to test the therapeutic effects of H_2_ on brain damage in patients resuscitated from cardiac arrest to obtain Pharmaceutical Affairs approval from the Ministry of Health, Labour and Welfare^8^. Apart from inhibiting ischaemia–reperfusion injury, H_2_ has been shown in animal studies to effectively suppress cytokine storms in a state of overreaction of the immune system. Notably, the Chinese National Health and Medical Commission recommends H_2_ inhalation in addition to general O_2_ therapy for treating coronavirus disease 2019 (COVID-19)-associated pneumonia or acute respiratory distress syndrome, and the effect of H_2_ inhalation in patients with COVID-19 was recently reported^9,10^.

The global prevalence of hypertension in adults is estimated to be 1.13 billion^11^. Hypertension is a strong risk factor for cardiovascular disease and chronic kidney disease, with hypertension observed in more than 80% of patients with chronic kidney disease^12,13^. As hypertension accelerates the decline in renal function and promotes the development of cardiovascular disease, proper management of hypertension in patients with chronic kidney disease is of paramount importance^14,15^. Although the importance of controlling blood pressure has been emphasised, more than 60% of patients who are hypertensive fail to adequately lower their blood pressure to the target level^16^.

Recently, Nakayama et al. developed a novel method to create an H_2_-enriched dialysate by reverse osmosis of H_2_ formed via the electrolysis of purified tap water, and demonstrated that compared to haemodialysis with standard dialysate, dialysis with H_2_-enriched dialysate improves blood pressure control in patients on chronic maintenance haemodialysis^17,18^. The influence of H_2_ on blood pressure was apparent only in patients with a post-dialysis systolic blood pressure higher than 140 mmHg.

Based on the evidence of this clinical trial, we investigated whether H_2_ exerts an anti-hypertensive effect in a rat model of hypertension.

## Results

### Development of a titrated H_2_ inhalation system

**Figure 1:**
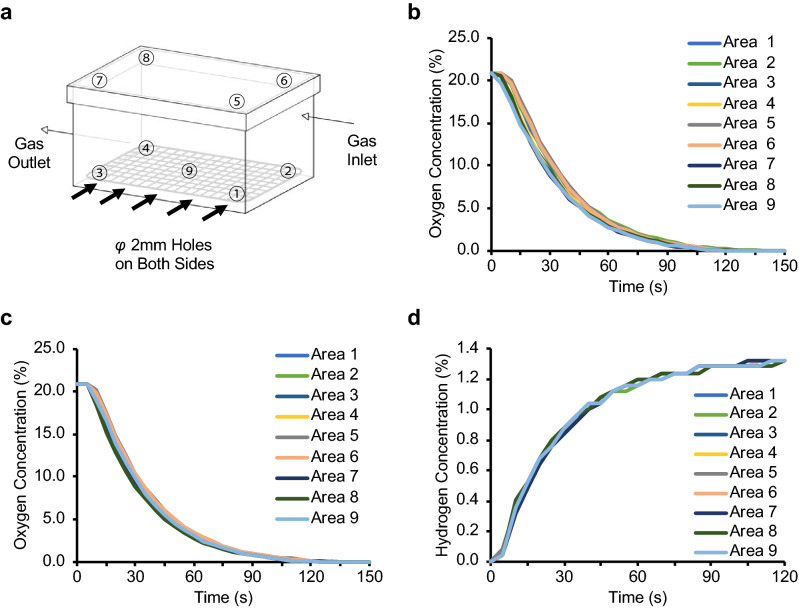
Schematic for developing a titrated H_2_ inhalation device. (**a**) A portable gas detector was placed at nine areas of an anaesthetic box (indicated by circled numbers) and the gas concentrations were measured. A total of 10 holes were drilled on two sides of the box to improve the homogeneity of the gas concentration. (**b**) Results of O_2_ concentration measurement before the holes were drilled. The concentration decreased faster at areas closer to the gas outlet. (**c**) Results of O_2_ concentration measurement after 10 holes were drilled on two sides of the box. With the holes, the decrease in the oxygen concentration became uniform among all nine areas. (**d**) H_2_ concentration after starting injection. H_2_ concentration increased uniformly in all areas and the air in the box was replaced by injected H_2_ after 2 min.

To determine the appropriate H_2_ flow rate, we estimated that the respiratory parameters of a rat were as follows: tidal volume, 2 mL; respiratory rate, 115 per minute; and expiratory CO_2_ concentration, 4%. Based on these parameters, we estimated the volume per minute as 0.23 L/min, with a CO_2_ excretion volume of 9.2 × 10^–3^ L/min. Stipulating the allowable CO_2_ concentration in the box to be less than 0.1% resulted in a calculated required gas flow rate of > 9.2 L/min; we therefore used a gas flow rate of 10 L/min. We tested the homogeneity of the gas flow in the box by flowing pure nitrogen gas at 10 L/min and measuring the O_2_ concentration at nine different locations. We found that O_2_ concentration decreased more rapidly near the outlet of the box (i.e. areas 3, 4, 7, and 8; Fig. 1a) than in the other areas (Fig. 1b). To resolve this inhomogeneity, we drilled holes on both sides of the box, which yielded a uniform rate of decrease in O_2_ concentration in all locations (Fig. 1c). In addition, approximately 2 min were required for the O_2_ concentration to reach 0%, indicating that at least 2 min are required for purging before a stable gas concentration is obtained. When the gas mixture of 1.3% H_2_ and 21% O_2_ in N_2_ was injected into the box, the H_2_ concentration was uniform in all areas, reaching 1.3% approximately 2 min from the start of gas flow (Fig. 1d).

### One-hour daily H_2_ therapy for 4 weeks suppresses blood pressure elevation in 5/6 nephrectomised rats

Rats were subjected to sham operation (Sham) or 5/6 nephrectomy (5/6 Nx). Post–5/6 Nx rats were randomised into two groups: 1-h daily exposure to H_2_ (5/6 Nx + H_2_) and control (control 5/6 Nx) for 4 weeks (Supplementary Fig. S1a). The one-stage 5/6 Nx conducted by a skilled surgeon using microsurgery yielded reproducible results with very little error, as evidenced by the fact that the levels of renal injury were uniform among individuals (Supplementary Fig. S1b–e). The two groups did not differ in the time course of changes in urine volume (Supplementary Fig. S1b), creatinine clearance (Supplementary Fig. S1c), blood urea nitrogen (BUN) (Supplementary Fig. S1d), creatinine (Supplementary Fig. S1e), or left kidney weight (Supplementary Fig. S1f.). Notably, the 5/6 Nx + H_2_ group showed faster postoperative weight recovery than the control 5/6 Nx (*P* = 0.08), although the difference in body weight between the two groups disappeared after 4 weeks (Supplementary Fig. S1g). The arterial blood pressure, measured with a pressure transducer placed at the right femoral artery 4 weeks after 5/6 Nx, was significantly lower in the 5/6 Nx + H_2_ group than in the control 5/6 Nx group (Supplementary Fig. S1h–j). These results suggest that 1-h daily H_2_ therapy can suppress the blood pressure increase after 5/6 Nx without affecting the level of renal impairment.

### Anti-hypertensive effect of H_2_ therapy occurs even with delayed treatment

**Figure 2:**
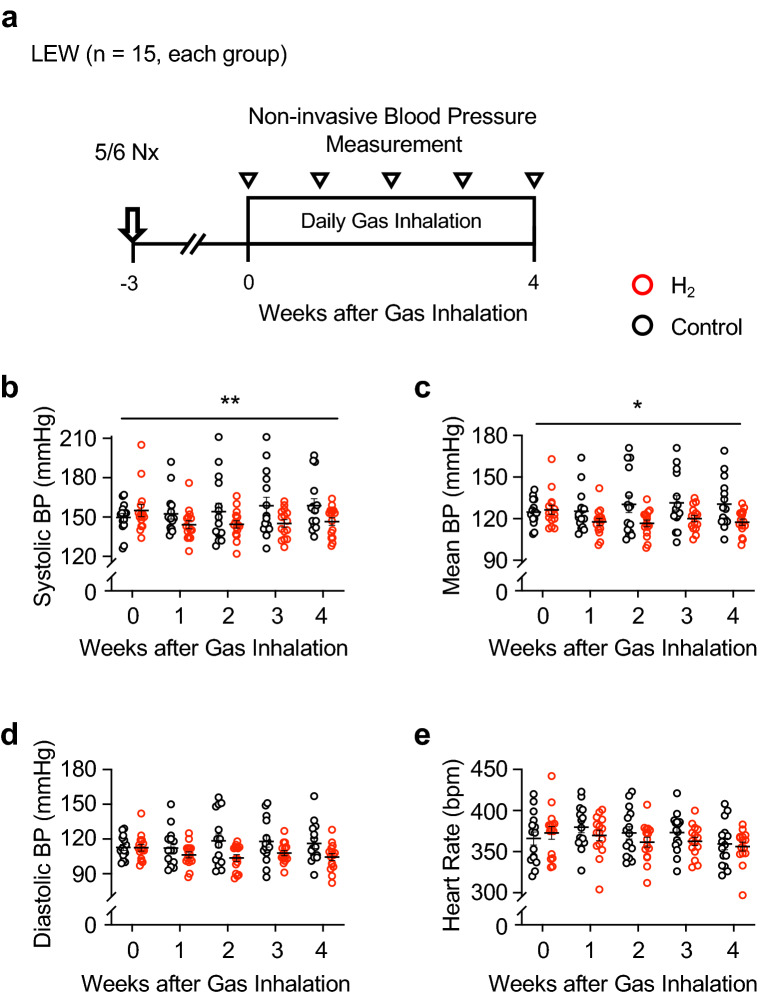
H_2_ therapy exerts an anti-hypertensive effect even when started 3 weeks after 5/6 nephrectomy. (**a**) Experimental protocol for measuring the effect of delayed administration of H_2_ on blood pressure in 5/6 nephrectomised (5/6 Nx) rats. 5/6 Nx was performed on rats (N = 34) 3 weeks prior to starting gas inhalation. Rats were randomly divided to H_2_ (N = 15) and control (N = 15) groups on the first day of gas inhalation (day 0). Four rats which did not develop hypertension were excluded from this experiment. Daily 1-h gas inhalation was continued for 4 weeks. (**b**) Systolic BP, (**c**) mean BP, (**d**) diastolic BP, and (**e**) heart rate. Haemodynamic parameters were measured weekly using the tail-cuff method (white arrowhead). *BP* blood pressure, *bpm* beats per min, *LEW* Lewis rats, *Nx* nephrectomy. Data are expressed as the mean ± SE. N = 15 in each group. Mixed effect model *P < 0.05, **P < 0.01.

Blood pressure may have decreased because H_2_ reduced inflammation associated with the nephrectomy. To test this hypothesis, we examined whether the blood pressure–lowering effect persisted even when H_2_ inhalation was started 3 weeks after 5/6 Nx. Blood pressure was measured weekly using a tail cuff. Thirty-four rats were subjected to 5/6 Nx; of these, 4 rats whose mean blood pressure did not increase to 105 mmHg or more 3 weeks after 5/6 Nx were excluded from subsequent experiments. The remaining 30 rats were randomly assigned to treatment and control groups (Fig. 2a). Four weeks of 1-h daily H_2_ therapy significantly lowered systolic and mean blood pressure compared with the control group (Fig. 2b–d). It also showed a tendency to decrease the heart rate, although the difference between the 2 groups was not significant (Fig. 2e).

### H_2_ alleviates autonomic nervous system imbalance in 5/6 nephrectomised rats

**Figure 3:**
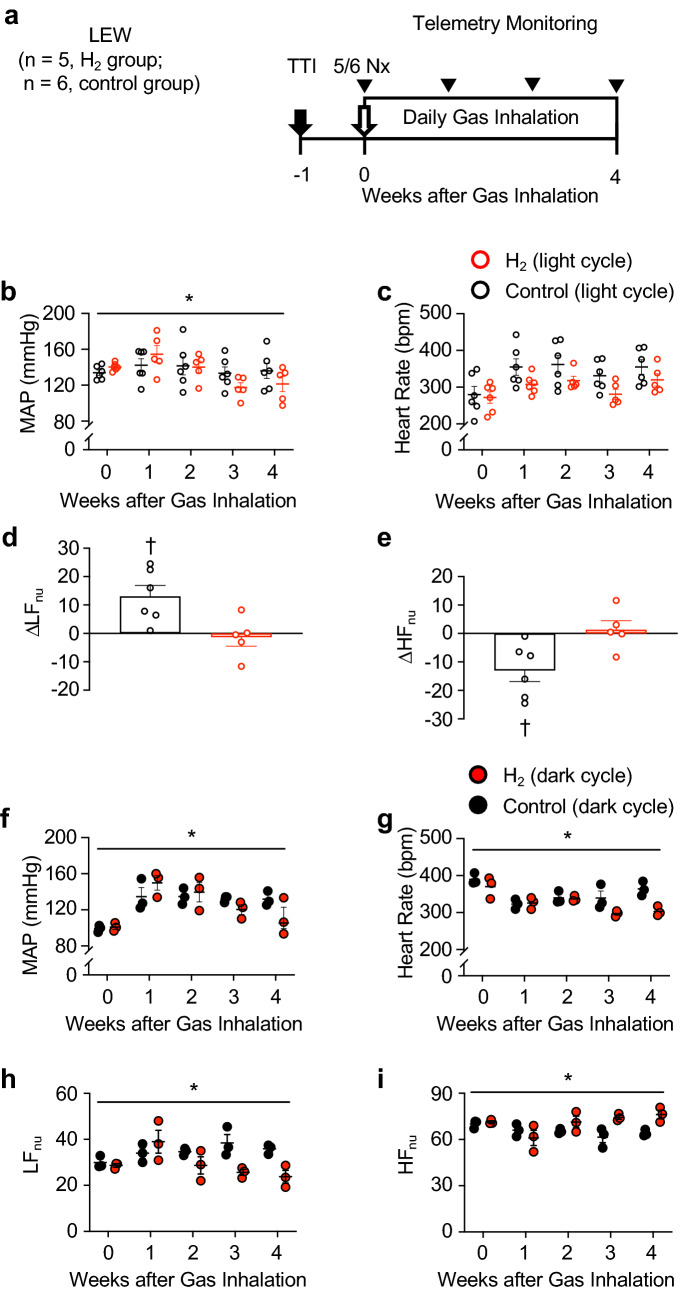
Anti-hypertensive effect of H_2_ is associated with an improvement in autonomic nervous system dysfunction. (**a**) Experimental protocol for non-invasive continuous blood pressure monitoring using wireless implantable telemetry in 5/6 nephrectomised (5/6 Nx) rats. The telemetry transmitter was implanted 1 week before the 5/6 Nx. Daily gas inhalation was initiated immediately after the 5/6 nephrectomy (Nx) (day 0) and was continued for 4 weeks. Haemodynamic monitoring was performed weekly using a telemetry system. The continuously monitored 60 min of blood pressure and heart rate, which were recorded 6 h after inhalation treatment was ceased, were used as resting-state daytime data. The 60 min recorded between 1:30 and 2:30 AM (the middle of the dark cycle) were used as the active-state night-time data. The day on which gas inhalation was initiated was defined as day 0. Thus, the daytime blood pressure on day 0 represents the blood pressure at 6 h after H_2_ inhalation, and the night-time blood pressure on day 0 represents the blood pressure between 1:30 and 2:30 AM before starting H_2_ inhalation. (**b–e**) Telemetry recordings of haemodynamic parameters during the light cycle. (**b**) Time course of change in mean arterial pressure (MAP) and (**c**) heart rate. (**d**) Change (Δ) in low-frequency (LF) power and, (**e**) high-frequency (HF) power from the first day to 4 weeks after nephrectomy. *LF*_*nu*_ low-frequency power in normalised units (nu), *HF*_*nu*_ high-frequency power in normalised units, *TTI* telemetry transmitter implantation. Data are expressed as the mean ± SE. Mixed effect model for MAP and heart rate analysis, *P < 0.05; paired-*t* test for blood pressure variability analysis, ^†^P < 0.05. (**f–i**) Telemetry recordings during the dark cycle. (**f**) Time course of change in MAP, (**g**) heart rate, (**h**) LF power, and (**i**) HF power. Mixed effect model, *P < 0.05.

To examine the anti-hypertensive effect of H_2_ in more detail, we conducted chronic and continuous monitoring of blood pressure using a non-invasive method with a wireless implantable telemetry system (Fig. 3a). Gas inhalation was started on the day of 5/6 Nx and continued for 4 weeks. Again, there was no difference in the time course of change in renal function between the two groups (Supplementary Fig. S2a,b). A longer period of gas inhalation led to a greater decrease in blood pressure in the 5/6 Nx + H_2_ group, compared to in the control 5/6 Nx group (P < 0.05) (Fig. 3b). As treatment continued, the trend towards a lower heart rate became more pronounced in the 5/6 Nx + H_2_ group, but the difference was not significant (Fig. 3c).

To examine the influence of H_2_ therapy on autonomic nervous system activity, we conducted spectral analysis of blood pressure variability. Spectral components were obtained in normalised units (nu). Low-frequency (LF) power indicates a predominantly sympathetic tone, whereas high-frequency (HF) indicates a mostly parasympathetic tone. Although the increase in LF power and decrease in HF power over time associated with 5/6 Nx showed a tendency to be suppressed by H_2_ inhalation, the results were not significant when compared as continuous variables over a 4-week time course (Supplementary Fig. S2c,d). In contrast, comparing the change from the baseline (day 0) to 4 weeks after starting H_2_ inhalation, the increase in LF power and decrease in HF power observed in the control 5/6 Nx group was significantly suppressed in the 5/6 Nx + H_2_ group (Fig. 3d,e).

Next, we examined whether 1-h daily H_2_ therapy affected blood pressure, heart rate, and autonomic nervous activity even during the nocturnal active period. We analysed 60 min of blood pressure and heart rate data recorded between 1:30 and 2:30, which is in the middle of the dark phase (from 21:00 to 7:00 of the next day). A longer treatment period led to greater reductions in blood pressure, heart rate, and LF power and increases in HF power in the 5/6 Nx + H_2_ group compared to in the control 5/6 Nx group (Fig. 3f–i).

## Discussion

This is the first study to experimentally demonstrate the pharmacological effects of H_2_ on hypertension in rats. We found that exposing 5/6 Nx rats to 1.3% H_2_ for 1 h per day for 4 weeks significantly suppressed increases in blood pressure due to 5/6 Nx.

H_2_ may have reduced blood pressure solely by mitigating local inflammation in the kidney caused by the 5/6 Nx. We tested this by repeating the experiment in rats that had fully recovered from surgical invasion. H_2_ inhalation treatment was started at 3 weeks after 5/6 Nx and continued for 4 weeks; blood pressure was measured weekly throughout this period with a tail cuff. Our results confirmed that H_2_ significantly reduced blood pressure in 5/6 Nx rats in the chronic phase, supporting that H_2_ has an anti-hypertensive effect independent of its anti-inflammatory properties during the perioperative phase.

To investigate the effect of H_2_ on blood pressure in more detail, we used an implanted telemetry system to continuously monitor blood pressure. This experiment revealed that H_2_ exerts its anti-hypertensive effect not only during daytime rest, but also during the nocturnal active phase. Our spectral analysis of blood pressure variability revealed that H_2_ mitigated the autonomic imbalance resulting from 5/6 Nx, namely by supressing the over-active sympathetic nervous system and augmenting parasympathetic nervous system activity; this effect was coincident with its blood pressure-lowering effects.

Our previous study suggested that inhaled H_2_ lowers blood pressure by acting directly on the brain. Most recently, we measured H_2_ dynamics in the blood of pigs after a single breath of H_2_^19^. Our experimental results showed that H_2_ was not simply diffused from the lungs but was efficiently transported through the arterial bloodstream to organs throughout the body, where it was dynamically metabolised. Inhalation of H_2_ increased the concentration of H_2_ in the carotid bloodstream to a higher concentration than anywhere else in the body. These observed dynamics for the H_2_ concentration in the blood after H_2_ inhalation suggested that its blood pressure–lowering effect was exerted via a direct impact on the brain.

Accumulating evidence indicates that hypertension in 5/6 Nx rats is mediated through increased sympathetic nerve activity elicited by the pathogenic interaction between the kidney and brain. It has been reported that the turnover rate of norepinephrine from the posterior hypothalamic nuclei was greater in 5/6 Nx rats than in control rats^20^. Bilateral dorsal rhizotomy has been shown to prevent increases in blood pressure and norepinephrine turnover in posterior hypothalamic nuclei^21^. These results indicate that increased renal sensory impulses are generated in the injured kidney and transmitted to the brain, where they excite vasomotor centres, which in turn activate efferent sympathetic nerve activity to the cardiovascular system and kidneys and lead to the development of hypertension^22,23^. Our results show that H_2_ exerted an anti-hypertensive effect without affecting the levels of 5/6 Nx–related renal dysfunction; this supports the conclusion that H_2_ acted directly on the brain rather than by reducing the generation of renal sensory impulses.

The therapeutic potential of H_2_ in humans is highly promising, but the research is still in its early stages. Anecdotal reports of H_2_ balancing the autonomic nervous system have been used in the sale of H_2_-related merchandise. H_2_ has been advertised to improve numerous conditions including autonomic neuropathy, insomnia, depression, stiff shoulders, headaches, loss of appetite, and stress-related diseases. None of these effects have been experimentally demonstrated; this is the first experimental study to show that H_2_ improves autonomic imbalance. However, these effects have not been demonstrated in humans, and the mechanism by which this effect is achieved remains unknown. Our preclinical research and clinical studies previously demonstrated that H_2_ reduces organ damage caused by ischemia–reperfusion in various medical emergency situations, including myocardial infarction^2,24^, haemorrhagic shock^5,25^, and out-of-hospital cardiac arrest^3,4^, as well as in organ transplantation^26^. Selective hydroxyl radical scavenging (•OH) has been widely accepted as the most likely mechanism of action of H_2_. Although H_2_ has been shown to selectively eliminate •OH in a test tube^27^, whether H_2_ reduces oxidative stress by eliminating •OH at the cellular and tissue levels remains unclear.

Activation of sympathetic nerves can lead to high blood pressure. Hirooka et al*.* showed that one brain region, the rostral ventrolateral medulla (RVLM), is important for the activation of sympathetic nerves, with a decrease in nitric oxide activity and increase in the production of ROS in RVLM as its possible mechanisms^28–30^. Campese et al*.* also showed that increased ROS production occurs in the brain during central sympathetic activation, which occurs during kidney injury^31,32^. H_2_ may suppress central sympathetic activity by scavenging ROS in the RVLM, thereby lowering the blood pressure and heart rate.

Although not the focus of our study, it is worth noting that postoperative H_2_ treatment reduced the degree of weight loss and promoted weight recovery after nephrectomy. Postoperative weight loss in humans contributes to poor prognosis by increasing the likelihood of complications and weakening the effectiveness of chemotherapy. Our results suggest that if these effects occur in humans, postoperative administration of H_2_ may shorten the length of hospital stays and improve the prognosis of patients after a variety of surgeries.

Our study has several limitations. First, insertion of the implanted telemetry system is a stressful procedure for rats and thus may have affected the variability in blood pressure and heart rate in. In addition, environmental factors such as sounds or odours^33^ may not have been completely eliminated when the measurements were taken. Third, further studies are needed to determine the optimum concentration of H_2_ and optimise the time of inhalation. Fine-tuning of the concentration of H_2_ required for different therapeutic outcomes will be required. Although concentrations of 1.3–4% have mostly been used in clinical trials relying on H_2_ inhalation, an increase in capillary blood flow has been observed by nail capillary microscopy even when inhaling much lower concentrations of H_2_ and after drinking H_2_-rich water. The amount of H_2_ required may vary depending on the biological effects being targeted. As this is the first study to investigate the therapeutic effects of H_2_ on hypertension, we used H_2_ with a sufficiently high concentration (1.3%) to ensure that any effect was detected. In fact, the maximum H_2_ concentration allowed under Japanese regulation to be added to a cylinder containing O_2_ is 1.3%, which is a third of the lower flammability limit of 4%. Our results revealed that at least 1 h of H_2_ inhalation per day was required to observe haemodynamic changes; however, whether inhalation of H_2_ for a longer time has a better effect should be further evaluated. Finally, additional studies are required to elucidate the versatility of H_2_ on blood pressure in other animal models of hypertension.

In conclusion, our study showed that daily 1-h H_2_ therapy lowered blood pressure in a rat model of hypertension and improved the imbalance of the autonomic nervous system caused by hypertension. Additional pre-clinical studies are required before the translational research.

## Methods

### Animals

Male Lewis rats (age, 8 weeks old; body weight, 250–300 g) were used (CLEA Japan, Tokyo, Japan). Animals were fed standard chow ad libitum with free access to water and were not fasted prior to the experiments. Animals were housed under standardised temperature (22 ± 1 °C) and humidity (55 ± 5%) conditions with a 14-h:10-h light: dark cycle. The rats were allowed to acclimatise to the above-mentioned conditions for a minimum of 1 week prior to the experiments. The study was approved by the Institutional Animal Care and Use Committee (Nippon Veterinary and Life Science University [Tokyo, Japan], No. 30 K-61; and Keio University [Tokyo, Japan], No. 13002-4). Allocation to the H_2_ and control groups was performed randomly. All animal experiments were performed in accordance with ARRIVE guidelines^34^.

### Development of H_2_ inhalation apparatus

We sought to establish a precisely titrated H_2_ inhalation system that simultaneously allowed gas inhalation and ambulatory blood pressure monitoring. We used an anaesthetic box (KN-1010 M, Natsume, Tokyo, Japan) for gas administration. The oxygen concentration was measured using a portable complex gas detector (GX-8000, Riken Keiki Co., Ltd., Tokyo, Japan) at nine areas in the box (Fig. 1a). Pure nitrogen gas (Taiyo Nippon Sanso Corporation, Tokyo, Japan) was flowed into the box at a rate of 10 L/min, and the oxygen concentration was recorded in the nine areas every 5 s until the oxygen concentration reached 0% v/v. To reset the experimental space, the box was opened for ventilation after each measurement, and measurements were repeated three times in each area. To achieve uniform filling of the box with gas, holes with a 2-mm diameter were drilled at five locations on both sides of the box to create a total of 10 holes, and the same test was repeated. Finally, 10 L/min of H_2_ gas (1.3% H_2_ + 21.0% O_2_ + 77.7% N_2_) (Taiyo Nippon Sanso Corporation, Tokyo, Japan) was flowed and H_2_ and O_2_ concentrations were measured at nine areas in the box using a GX-2009 detector (GX-2009, Riken Keiki Co., Ltd., Tokyo, Japan). We confirmed that the inside of the box was uniformly filled with the mixed gas.

### Gas inhalation

Gas cylinders for injection into the anaesthetic box were filled with H_2_ gas (1.3% H_2_ + 21.0% O_2_ + 77.7% N_2_) and control gas (21.0% O_2_ + 79.0% N_2_) at a factory (Taiyo Nippon Sanso Corporation, Tochigi, Japan). The H_2_ group and control group inhaled the premixed H_2_ gas and control gas, respectively. The gas flow rate was 10 L/min and the animals were kept in the box for 1 h at a time. An additional 3 min was set for purging the box (i.e. 63 min in total). In all experimental protocols, the day on which the gas inhalation was initiated was uniformly defined as day 0. The treatment was repeated every day for 4 weeks.

### Surgical procedure for partial nephrectomy

To induce renal hypertension, 5/6 nephrectomy was performed as previously described^35–37^, with minor modifications. Briefly, the rats were anaesthetised via isoflurane inhalation (induction at 4%, maintained at 1.5%), and an upper median laparotomy of approximately 4 cm was performed. Surgical procedures were carried out under a microscope; the branches of the left renal artery were identified and then selectively ligated with 7–0 silk sutures as close to the left kidney as possible to grossly infarct approximately 2/3 of the left renal cortex. Subsequently, the right renal artery, renal vein, and ureter were ligated with 4–0 silk sutures and the right kidney was resected. The abdominal wall and skin were closed with 4–0 nylon thread. Sham-operated rats were subjected to the same laparotomy as 5/6 Nx except that surgical interventions to the kidneys was not performed. The anaesthesia time was 30 min, which was uniform for all rats.

### Blood pressure measurement

In the first experiment, arterial blood pressure was measured at the right femoral artery using a pressure transducer (PE50, Natsume, Tokyo, Japan; DX-360, Nihon Kohden, Tokyo, Japan) after 4 weeks of gas inhalation under anaesthesia induced by isoflurane inhalation. In the second experiment, blood pressure and heart rate were measured by the tail cuff manometry (BP-98A, Softron, Tokyo, Japan) method. Blood pressure and heart rate were measured in triplicate, and the median value was used as the representative value for that individual.

### Implantation of telemetry transmitter

Rats were anaesthetised using isoflurane and the left groin was disinfected with 1% chlorhexidine. An incision of approximately 1.5 cm was made and the left femoral artery was exposed. A telemetric transmitter (HD-S10, Physiotel HD Telemetry, Data Science International, St. Paul, MN, USA) catheter was inserted into the left femoral artery^38^. The tip of the transmitter catheter was placed in the abdominal aorta caudally from the renal artery bifurcation. The transmitter body was inserted into a subcutaneous pocket created on the left lower back, and the skin was sutured. All surgical interventions were performed in an aseptic manner.

### Continuous haemodynamic parameter monitoring

Blood pressure and heart rate were continuously monitored using a telemetry system (Ponemah Ver. 6.3, Data Science International, St. Paul, MN, USA). Data were recorded for 60 min in a breeding cage. Blood pressure data obtained at 1 and 6 h after stopping inhalation were used for analysis. If this measured value was a significant outlier compared to the other measurements of the same individual on the same day, the data obtained from the hour before inhalation were used instead. Of the recorded data, those acquired within the first 15 min of each measurement period were excluded from analysis. Data retrieved in the remaining 45 min were divided into 5-min blocks. Blood pressure and heart rate display great fluctuations when the rats move; thus, in each 5-min data block, the values in the first 1 min of stable measurement were recorded and averaged. Stable measurement was defined as data without body movement and without any parts missing due to loss of signal; if these conditions were not met, the data were considered as invalid. Within each 5-min block, if the data of the first minute was deemed as invalid, data from the following minute were used. In the rare cases in which all single-minute intervals within the 5-min block were invalid, the data from that block were discarded completely.

### Spectral analysis of arterial blood pressure variability

Arterial blood pressure variability was analysed using a telemetry system software (Ponemah Ver. 6.3, Data Science International, St. Paul, MN, USA). Frequency domain analysis (500 Hz sampling rate; very low frequency [VLF], 0.05–0.25 Hz; LF, 0.25–1.0 Hz; HF, 1.0–3.0 Hz) was performed using arterial pressure waveform data of the first minute (approximately 300–400 beats) every 5 min. Similar to the method employed for evaluating haemodynamic parameters, in case there was a body movement or any missing data due to loss of signal, the next minute was used, and if all single-minute intervals of the 5-min block were invalid, data from that block were discarded.

### Renal function measurement in 5/6 nephrectomy rats

Renal function was assessed before, one day after nephrectomy, and weekly by using a handheld blood analyser (i-STAT cartridge CHEM8 + , Abbott Japan, Chiba, Japan; i-STAT analyser, Abbott, Chicago, IL, USA). Daily urine volume was measured weekly using a metabolic cage (KN-646, Natsume, Tokyo, Japan) and creatinine clearance (mL/min/kg BW) was calculated. The left kidney weight was measured at the end of the experiment after 4 weeks of gas inhalation.

### Statistical analysis

Descriptive statistics are presented as the mean ± standard error of mean. Comparisons were conducted using analysis of variance, unpaired *t*-test, paired *t*-test, or Mann–Whitney U-test, as appropriate. The Tukey’s multiple comparison test was followed by analysis of variance. The mixed-effect model was used to analyse the repeated measures. All tests were two-tailed, and a P value < 0.05 was considered as statistically significant. All statistical analyses were conducted using GraphPad Prism 8.0 (GraphPad Software, Inc., La Jolla, CA, USA).

## Supplementary information

Supplementary Information

## Data Availability

The datasets generated and/or analysed during the current study are available from the corresponding author upon reasonable request.
