# Supplementary material for: Daily inhalation of hydrogen gas has a blood pressure-lowering effect in a rat model of hypertension
Source: Sci Rep. 2020 Nov 26;10:20173. doi: 10.1038/s41598-020-77349-8 (PMC7692487; doi:10.1038/s41598-020-77349-8)
Supplement: Supplementary file 1 — Supplementary Information [file 41598_2020_77349_MOESM1_ESM.pdf]

Supplementary materials

Daily inhalation of hydrogen gas has a blood pressure–lowering effect in a rat model of hypertension

Sugai K, Tamura T, Sano M, Uemura S, Fujisawa M, Katsumata Y, Endo J, Yoshizawa J, Homma K, Suzuki M, Kobayashi E, Sasaki J, and Hakamata Y

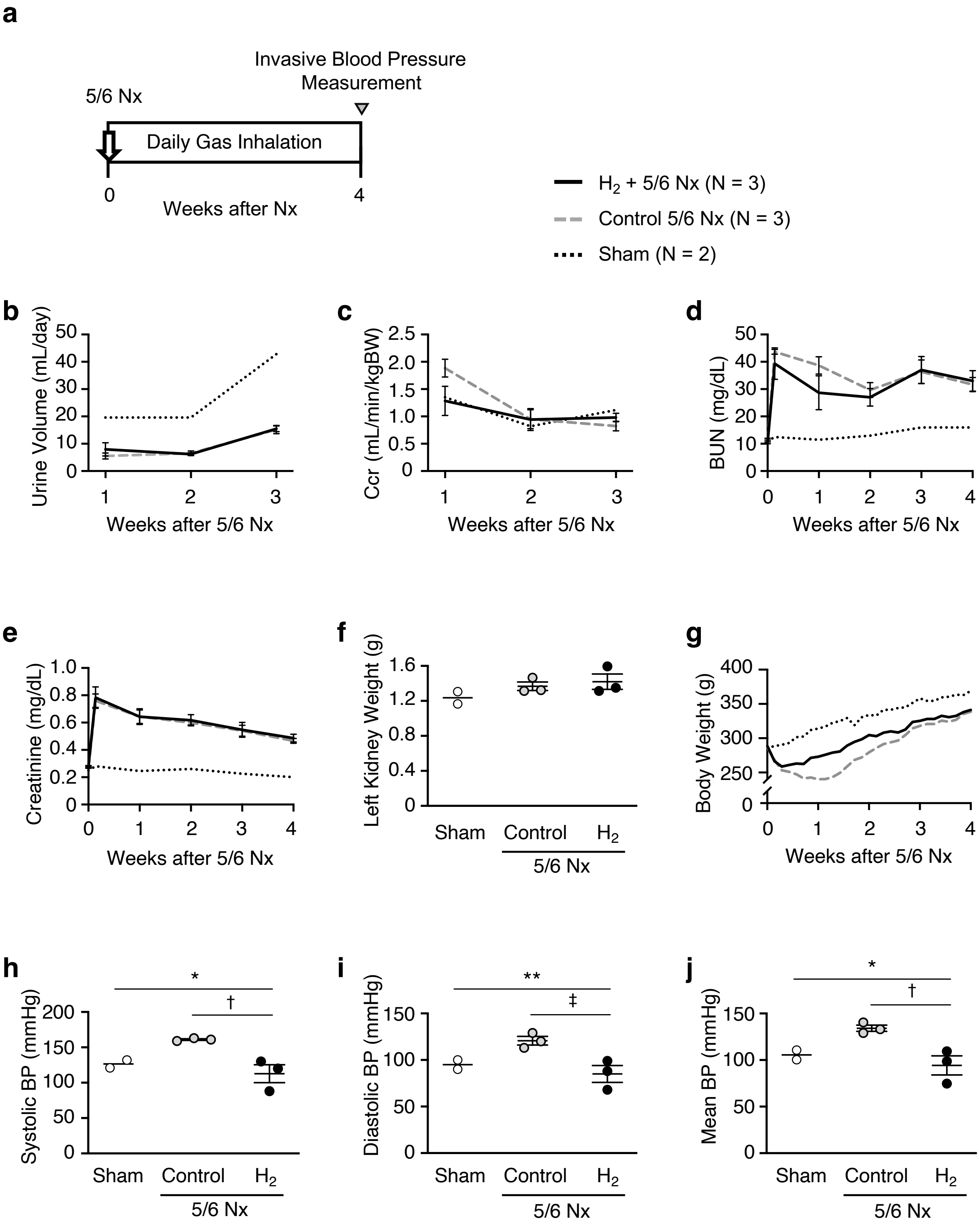

Supplementary Fig. S1. One-hour daily H_2 therapy suppresses blood pressure increase after 5/6 nephrectomy. **a)** Experimental protocol for measuring the effect of early administration of H_2 on the blood pressure of 5/6 nephrectomised (5/6 Nx) rats.

The groups measured using this protocol were as follows: control gas–treated sham-operated (Sham) rats, control gas–treated 5/6 Nx (control 5/6 Nx) rats, and H_2-treated (5/6 Nx + H_2) rats. Gas inhalation treatment was started immediately after respective surgery (day 0). After 4 weeks, a pressure transducer was placed at the right femoral artery and arterial blood pressure was measured under inhaled anaesthesia. **b)** Change in urine volume, **c)** creatinine clearance (Ccr), **d)** blood urea nitrogen (BUN) level, **e)** creatinine level, **f)** left kidney weight, and **g)** body weight change after Nx. **h)–j)** Comparison of blood pressure (BP) parameters (systolic BP; diastolic BP; and mean arterial pressure [MAP]) 4 weeks after surgery. Data are expressed as the mean ± SE. N = 3 in each group. ANOVA; * $P < 0.05$, ** $P < 0.01$. Tukey's multiple comparison test; † $P < 0.05$, ‡ $P < 0.01$.

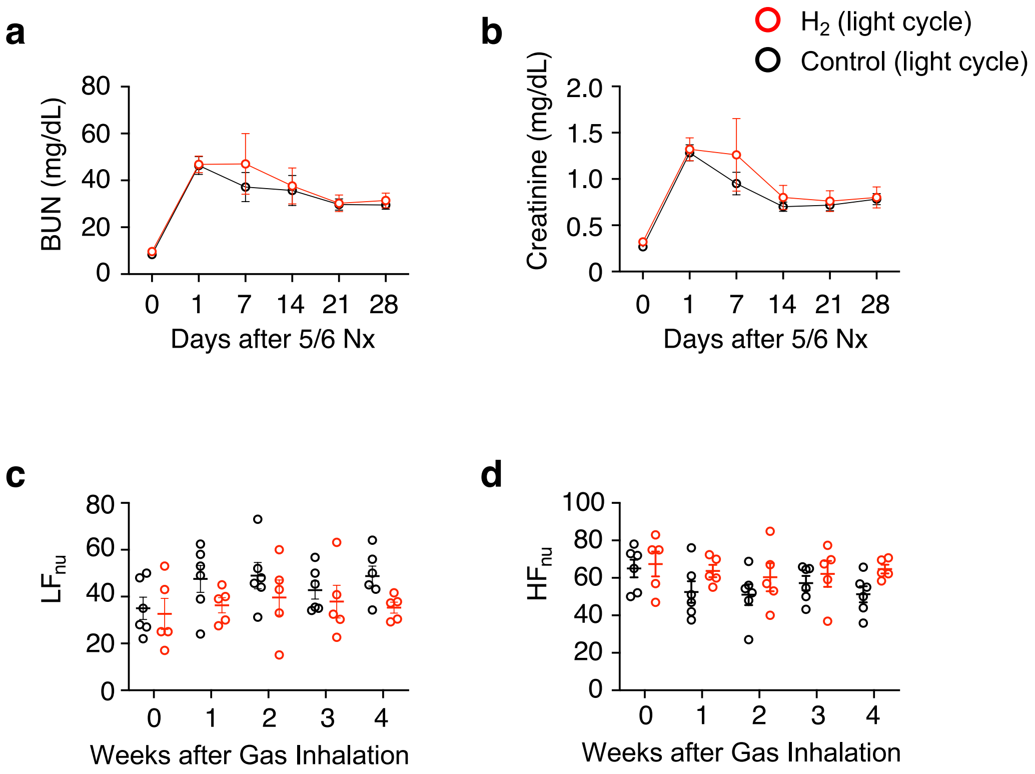

Supplementary Fig. S2. Time course change in renal and autonomic function following 5/6 nephrectomy. Blood pressure and heart rate were recorded at 6 h after the end of gas inhalation treatment. BUN, blood urea nitrogen; HF_{nu}, high-frequency power in normalised units; LF_{nu}, low-frequency power in normalised units; Nx, nephrectomy. Data are expressed as the mean ± SE. N = 5 and N = 6 for H_2 and control group, respectively.
